# Adult Hippocampal Neurogenesis in Parkinson's Disease: Impact on Neuronal Survival and Plasticity

**DOI:** 10.1155/2014/454696

**Published:** 2014-07-03

**Authors:** Martin Regensburger, Iryna Prots, Beate Winner

**Affiliations:** ^1^IZKF Junior Research Group and BMBF Research Group Neuroscience, IZKF, Friedrich-Alexander University of Erlangen-Nuernberg (FAU), Glückstraße 6, 91054 Erlangen, Germany; ^2^Department of Neurology, Friedrich-Alexander University of Erlangen-Nuernberg (FAU), Schwabachanlage 6, 91054 Erlangen, Germany

## Abstract

In Parkinson's disease (PD) and other synucleinopathies, chronic neurodegeneration occurs within different areas of the central nervous system leading to progressive motor and nonmotor symptoms. The symptomatic treatment options that are currently available do not slow or halt disease progression. This highlights the need of a better understanding of disease mechanisms and disease models. The generation of newborn neurons in the adult hippocampus and in the subventricular zone/olfactory bulb system is affected by many different regulators and possibly involved in memory processing, depression, and olfaction, symptoms which commonly occur in PD. The pathology of the adult neurogenic niches in human PD patients is still mostly elusive, but different preclinical models have shown profound alterations of adult neurogenesis. Alterations in stem cell proliferation, differentiation, and survival as well as neurite outgrowth and spine formation have been related to different aspects in PD pathogenesis. Therefore, neurogenesis in the adult brain provides an ideal model to study disease mechanisms and compounds. In addition, adult newborn neurons have been proposed as a source of endogenous repair. Herein, we review current knowledge about the adult neurogenic niches in PD and highlight areas of future research.

## 1. Unmet Needs in the Treatment of Parkinson's Disease

Parkinson's disease (PD) is an age-related chronic neurodegenerative disorder with an estimated prevalence of 160 per 100,000 affecting 2-3% of people aged 55 and above [1, 2]. The clinical diagnosis is based on the presence of the motor symptoms bradykinesia, resting tremor, rigidity, and postural instability [3], while the definitive diagnosis can only be made* post mortem* by detection of *α*-synuclein containing Lewy bodies (LB) in the substantia nigra (SN) [4]. A number of therapies are available to alleviate the motor symptoms including L-DOPA (as gold standard), dopamine agonists, MAO-B-inhibitors, the NMDA-receptor-antagonist amantadine, and neuromodulation by deep brain stimulation. However, none of them has proven disease-modifying effects and the clinical benefits of the therapy may wear off as the disease progresses [5]. Cell replacement strategies to replace lost dopaminergic input in the striatum of PD patients have led to the proof of principle that fetal mesencephalic transplantations into the striatum increase striatal dopamine levels [6, 7] and reinnervate the striatum [6, 8–13] but came to a first stop when severe graft induced dyskinesias were found as a major complication [14, 15]. Moreover, detailed analysis of the tissue revealed signs of host-to-graft propagation of LB pathology [10, 12]. Recently, a new trial has been instated that aims at reviving and refining the technique and is funded by the EU as the multicenter project TRANSEURO [16].

Nonmotor symptoms of PD have gained increasing interest due to their major impact on the patients' quality of life and due to the limited availability of symptomatic treatments [17–19]. Indeed, the variety of nonmotor symptoms reflects the multisystemic nature of PD, according to the current concept of disease propagation in an ascending pattern [3, 20]. Affected brain regions are identified first by *α*-synuclein-positive LBs and dystrophic Lewy neurites (LN) [21]. LB deposition is accompanied and followed by neurodegeneration, but the processes that precede this stage are unclear as yet. According to the staging proposed by Braak and colleagues, PD pathology is only detected in two basal cranial nerves nuclei, namely, the glossopharyngeal and the vagal nerve and in the olfactory bulb at an early disease stage (OB, stage 1). This corresponds to the premotor symptoms hyposmia and autonomic dysfunction, including obstipation [22–25]. At stage 2, pathology is detectable also in the pontine areas of the locus coeruleus, the raphe nuclei, and the reticular formation. This brainstem affection may cause rapid-eye-movement sleep behavior disorder which is one of the most specific indicators for the future development of PD and occurs in 30–50% of PD patients [26, 27]. Depression is a nonspecific but frequent nonmotor symptom of PD that often begins in the prodromal phase and severely affects the quality of life in PD [28, 29]. Stage 3 of PD histopathology marks the involvement of the SN and the anterior olfactory nucleus, whereas significant rates of degenerating neurons in the pars compacta of the SN are only seen in stage 4. Motor symptoms of PD emerge at stage 4 or later, when disease pathology is already widespread and a substantial proportion of SN neurons degenerated. Thus, disease-modifying therapies should be much more promising when instated in early premotor stages of PD. To this end, PD risk scores have been introduced [30, 31]. Stages 5 and 6, finally, are characterized by the involvement of the basal forebrain and cortical regions, including the entorhinal cortex and the cornu ammonis regions of the hippocampus. This advanced stage of PD is clinically dominated by complicated control of motor symptoms (e.g., fluctuations, dyskinesias, and dysphagia) and severe nonmotor symptoms like Parkinson's disease dementia (PDD), psychosis, and sleep-wake disorders. Dementia with Lewy bodies (DLB) is characterized clinically by a predominant dementia syndrome preceding motor symptoms and pathologically by neocortical accentuation of LB pathology [32–34].

In light of the chronically progressive disease pattern of PD involving olfactory and hippocampal systems, the presence of neural stem cells and active neurogenesis throughout life serves as an attractive model to study PD disease pathology and to test neuroprotective and neuroregenerative treatment approaches. Therefore, in the following review, we will elaborate current knowledge about adult neurogenesis in PD patients and PD models, and we will discuss how these findings may help to understand and to treat PD.

## 2. Adult Neurogenesis in PD Patients

It is accepted today that neurogenesis persists in humans in the dentate gyrus (DG) of the hippocampus and in the subventricular zone (SVZ) beyond embryonic neurogenesis [35–38]. Few studies have addressed adult neurogenesis in PD patients, mainly in the SVZ/OB system. Small and heterogeneous sample groups, post-mortem delay, and the availability of immunohistochemical markers have limited the direct investigation of alterations of adult neurogenesis in the two neurogenic zones of PD patients. The number of cells positive for proliferating cell nuclear antigen (PCNA) was reduced in the SVZ of 4 PD patients when compared to 4 controls [39]. This proliferation defect was related to reduced dopaminergic innervation from the SN pars compacta as seen in animal models of dopaminergic deafferentation [39–42]. In line with the hypothesis of dopaminergic control of SVZ proliferation, a decreased number of epidermal growth factor (EGF) receptor positive cells were found in the SVZ of 6 PD patients as compared to 6 age- and sex-matched controls [43]. In a clinicohistological study of a cohort of 32 PD patients, the number of Musashi-positive cells within the SVZ (representing neural stem and progenitor cells within this area) was positively correlated with the extent of dopaminergic treatment whereas disease duration showed a negative correlation [44]. A similar reduction of Musashi-positive cells within the SVZ was noted in specimen of 5 DLB and 6 PDD cases as compared to 5 controls [45]. The observed decrease in SVZ proliferation of patients with LB disease may thus be due to a reduction of the number of putative stem cells as a consequence of dopamine depletion. However, a recent study did not detect changes in SVZ proliferation (as determined by expression of PCNA and pHH3) in post-mortem* tissue* of 10 PD patients when compared to 10 controls [46]. No changes in the number of GFAP*δ*-positive cells as another putative marker of SVZ stem cells were observed [47]. This study controlled for age, sex, and post-mortem delay and included additional specimen from 5 nondemented controls with incidental LB pathology to take dopaminergic treatment into account. The authors did not detect differences of SVZ proliferation in the presence of high intragroup variability. The ability to generate SVZ-derived cultures from PD post-mortem tissue provides a hint for its intact proliferative capacity but currently does not allow quantitative conclusions [46, 48]. Within the authors' explanations for the discrepancies between these human SVZ studies, it became clear that consent about the optimal methodology concerning tissue sampling, anatomical definition of sampling area, choice of markers for SVZ stem and progenitor cells, and quantification still needs to be defined [49, 50]. Future studies with new methods thus need to be designed and carefully conducted to resolve these conflicting data.

There is a strong correlation between nigral dopaminergic degeneration, cholinergic deficits within the limbic system, and the premotor symptom hyposmia as shown by imaging data [51, 52]. Odor discrimination is a hippocampus-dependent task [53] and hippocampal dopaminergic hypoactivity correlates with hyposmia in PD [54]. However, in addition to the early neuropathological involvement of the OB during the course of PD, direct studies about alterations of the OB in PD suggest that pathogenesis of hyposmia may also take place within the OB. The OB volume was found to be reduced in a post-mortem study of 7 PD patients and 7 controls [55]. Other studies, however, found unchanged OB volumes in PD patients on MR-imaging [56] and histopathologically along with an increased number of dopaminergic OB neurons [57, 58]. In summary, currently available data suggest a complex pathogenesis of hyposmia in PD involving the OB and potentially secondary brain structures.

With regard to hippocampal neurogenesis, the density of nestin- and beta3-tubulin-positive cells was found to be reduced in the DG of 3 patients with PD and in the DG of 5 patients with PDD when compared to 3 controls [39]. Similarly, in the DG of 6 patients with DLB, the number of Sox2-positive putative stem cells was decreased, as compared to 6 controls [59]. The involvement of the hippocampus in nonmotor symptoms of PD has gained increasing attention. Fatigue and depression have been related to hippocampal serotonergic dysfunction by positron emission tomography with specific metabolites of serotonergic metabolism [60, 61]. Besides, the hippocampus is modulated by dopaminergic input from the ventral tegmental area and the olfactory bulb and by noradrenergic input from the locus coeruleus and may thus be involved in drive and mood regulation [62]. Cognitive deficits in PD are heterogeneous and have mainly been implicated in cholinergic and noradrenergic dysfunction involving hippocampal functions (reviewed in [63]). The extent of hippocampal LB pathology correlated with the degree of dementia in PD patients [64]. Significant hippocampal atrophy is seen on magnetic resonance imaging of patients with PDD when compared to nondemented PD patients (reviewed in [65]). Alterations of hippocampal connectivity by diffusion tensor imaging in PD patients predicted the emergence of declarative memory deficits [66] indicating that altered plasticity may be one of the reasons for structural changes. In summary, hippocampal dysfunction is common in PD patients and likely contributes to depression and cognitive impairment. As both of these nonmotor symptoms have been related to defects in adult neurogenesis, more research about human hippocampal neurogenesis in PD is needed to prove a causal role.

## 3. Lesion Models of PD

In contrast to the limited amount of data and material from human PD brains, many studies have been conducted in PD animal models, mainly in rodents. Stereotactic delivery of 6-hydroxydopamine (6-OHDA) into the SN or the medial forebrain bundle leads to lesions of the striatonigral pathway and thus replicates the striatal dopaminergic deficit [67]. Different studies have shown a negative impact of dopaminergic deafferentation on neural progenitor cell (NPC) proliferation, probably due to decreased input via D2L-receptors [39, 40, 42, 43, 68]. Despite a decrease in SVZ proliferation in the 6-OHDA lesion model, the number of newly generated dopaminergic neurons in the glomerular layer of the OB is increased, paralleling the finding of higher numbers of dopaminergic glomerular neurons in the OB of PD patients [42]. Dopaminergic stimulation increases proliferation in nonlesioned and lesioned rodents [43, 69]. Local application of the growth factors EGF and FGF-2 not only enhances SVZ proliferation but also induces striatal migration of NPCs [70–72]. In contrast to the aforementioned results, two studies found increased SVZ proliferation upon 6-OHDA lesion [73, 74].

Systemic administration of 1-methyl-4-phenyl-1,2,3,6-tetrahydropyridine (MPTP) is another way of ablating dopaminergic neurons via mitochondrial damage. Acute administration of high doses results in decreased SVZ proliferation along with an increased rate of apoptosis of migrating neuroblasts [39, 75–77] which was confirmed in a study in nonhuman primates [41]. In contrast, another group reported increased rates of proliferation in the acute MPTP model [78, 79] and chronic MPTP-treatment at lower doses did not alter SVZ proliferation [46].

In summary, a definite statement about the precise effect of dopaminergic lesions on SVZ proliferation cannot be made, but a negative effect of dopaminergic depletion on SVZ proliferation was a common finding. As mentioned in the previous chapter, it remains disputable whether this also holds true for PD patients.

## 4. *α*-Synuclein Transgenic Models of PD

Lesion models acutely damage dopaminergic structures and result in a pronounced motor phenotype. Transgenic models of PD exhibit milder but chronically progressive deficits including nonmotor symptoms (reviewed by [80–83]). In addition, transgenic models provide an insight into the disease mechanism of relevant genes and proteins.

The protein *α*-synuclein has been causally linked to PD. It is present in LB and LN [21, 84, 85] and different *α*-synuclein mutations and duplications and triplications cause genetic PD [86–92]. Common single nucleotide polymorphisms in the *α*-synuclein locus are significantly associated with PD [93, 94].

Adult neurogenesis has been studied in different *α*-synuclein transgenic animal models. A transgenic mouse model overexpressing human wild-type *α*-synuclein under control of the PDGF*β* gene promoter exhibits widespread accumulation within the central nervous system including the hippocampus along with age-dependent memory deficits [95, 96]. In these mice, the survival of newborn neurons is compromised both in the hippocampus and in the olfactory bulb, paralleled by increased levels of cell death in these regions [97]. In mice overexpressing the familial *α*-synuclein mutant A53T under the same promoter, the neurogenesis deficit is even higher indicating increased toxicity due to the mutation [98, 99].

In a different transgenic mouse model of synucleinopathies, *α*-synuclein is overexpressed under tetracycline-regulatable control of the CaMKII*α* promoter which led to neurodegeneration within the SN and the hippocampus [100]. Similar to the PDGF*β* promoter transgenic animals, the survival of newborn neurons is impaired in the hippocampus and in the olfactory bulb of these animals and in conditional overexpressers of A30P-mutant human *α*-synuclein [100–103]. Interestingly, transgene repression reversed the neurogenic deficit in overexpressers of wild-type *α*-synuclein in the hippocampus, but only partially in the OB. In contrast, transgene repression in A30P-mutant *α*-synuclein transgenic mice reversed the neurogenic deficit in the olfactory bulb, but not in the hippocampus. In principle, the partial restoration of the neurogenic deficit indicates a survival deficit at the integration site rather than a persisting developmental defect and proves in principal that *α*-synuclein pathology is reversible. The persisting deficit of hippocampal neurogenesis despite transgene repression in A30P-mutant *α*-synuclein transgenic animals was related to the uptake of *α*-synuclein from neurons into glial cells [102]. In addition, the A30P mutation shows a higher propensity to form oligomers [104]. *α*-Synuclein toxicity in general may be mediated by a prion-like propagation. *α*-Synuclein is released into the extracellular space and can be taken up by neurons, NPCs, and astrocytes [105–107]. This may explain the continuous spread of pathology in PD [20] and has to be kept in mind when planning transplantation strategies [108, 109]. The finding that glial A30P-*α*-synuclein is not cleared upon transgene repression suggests that *α*-synuclein propagated into glial cells persistently impairs the integration of newborn neurons independent of cell-autonomous expression of *α*-synuclein within the neuron itself [102].

In a BAC-transgenic rat model, human *α*-synuclein gene was expressed with its whole genomic locus [110]. These rats exhibited early behavioral changes and a subsequent progressive motor phenotype along with a marked decrease of striatal dopamine content and nigral degeneration. The number of newborn neurons in the glomerular layer of the OB (mostly dopaminergic neurons) was increased in these rats, paralleling preliminary results in humans and the 6-OHDA lesion mouse model [42].

The mechanisms that lead to defective neurogenesis in *α*-synuclein transgenic animals are still not well understood. Transgenic overexpression of *α*-synuclein is accompanied by decreased levels of Notch which may be mediated by increased p53 signaling [111, 112].

There is growing evidence that oligomeric forms of *α*-synuclein rather than LB and LN constitute the toxic species in the process of *α*-synuclein aggregation [113]. Interestingly, an artificial mutant of *α*-synuclein that is highly prone to form oligomers causes increased dopaminergic toxicity within the SN and synaptic loss in a transgenic mouse model [114, 115]. Therefore, the specific effect of oligomeric species on newborn neurons may be of interest to study pathogenic events of synaptic integration in the future.

**Figure 1:**
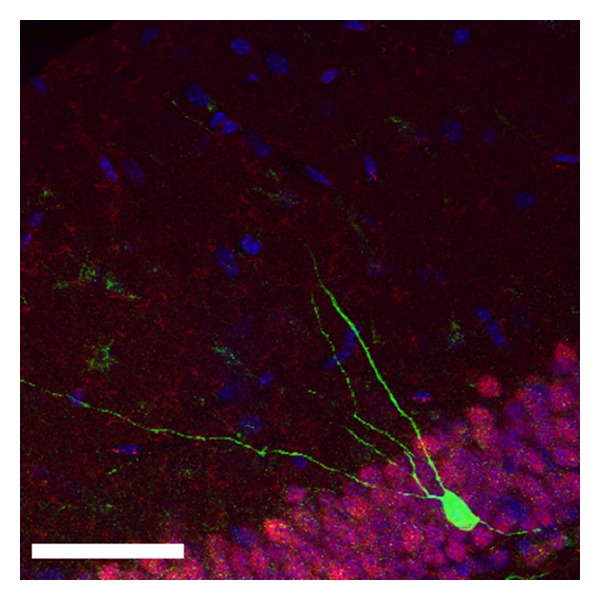
Newborn neuron in the hippocampus of an *α*-synuclein transgenic mouse labeled retrovirally with GFP.* Scale bar* 50 *μ*m.

When studying survival of adult newborn neurons, one has to keep in mind that a complex process of migration, phenotypic transition, lineage determination, outgrowth, and synaptic integration is involved, modulated by many different stimuli [116]. The survival of newborn neurons depends on their proper integration and on a certain degree of synaptic input activity (reviewed in [117]). The outgrowth of dendrites and the formation of synaptic spines are prerequisites for synaptic input. Indeed, dendritic morphology of adult newborn neurons is significantly reduced in *α*-synuclein transgenic animals [59] with an example shown in Figure 1. In addition, the density of mushroom spines reflecting stable synaptic input onto newborn neurons is reduced in these animals. Therefore, increased cell death and reduced survival of newborn neurons in *α*-synuclein transgenic animals may be due to defects in outgrowth and synaptic integration. Upon cell-specific overexpression of *α*-synuclein in newborn neurons, dendrite outgrowth but not mushroom spine formation was decreased. This led to the conclusion that the dendritic outgrowth defect is due to cell-autonomous effects of *α*-synuclein. *α*-Synuclein may, for example, interfere with dendritic outgrowth by direct interaction with microtubule associated proteins and thereby disrupt microtubule assembly and transport [118]. The cAMP response element-binding protein (CREB) pathway could also play a causal role since its activation by the phosphodiesterase inhibitor rolipram rescued the outgrowth defect [59]. Dystrophic LNs are a common feature of PD pathology, but they represent a final stage of neuritic degeneration and *α*-synuclein aggregation [20, 119]. The defect of dendrite growth and spine formation rather represents an early feature of PD pathology. Indeed, synaptic dysfunction is an early feature in synucleinopathies and is accompanied by loss of dendritic spines [120–122]. Transgenic overexpression of *α*-synuclein alters the vesicle composition of the synapse (“vacant synapse”) and leads to neurotransmitter release deficits [123]. Thus, the neurogenic system in *α*-synuclein transgenic animal models represents certain features of PD pathology and therefore constitutes a model to study the effect of drugs on synaptic pathology and cellular survival in PD.

It is still unclear whether the physiological function of *α*-synuclein overlaps with its pathogenic effects in PD [124]. *α*-Synuclein was originally described as a modulator of plasticity and neurogenesis during songbird learning [125]. Knockout of *α*-synuclein in mice does not lead to an overt phenotype, but rather to minor changes of dopaminergic neurotransmission, especially when *β*-synuclein is also deleted [126, 127]. In line with this, *α*-synuclein knockdown by RNA-interference in hippocampal neurons reduces the presynaptic vesicle pool size [128]. Physiologically, *α*-synuclein also exerts neuroprotective functions, since deletion leads to increased vulnerability to cysteine-string protein-*α* (CSP*α*) deletion and to nigral cell death [129, 130]. Therefore, it is not surprising that neurogenesis is altered in *α*-/*β*-synuclein double-knockout mice [59]. Neuronal differentiation of newborn neurons was increased, which may be caused by altered dopaminergic signaling inputs from the perforant path [131]. Overexpression of human *α*-synuclein in newborn neurons in the *α*-/*β*-synuclein-null background does not impair dendrite outgrowth [59]. This suggests that a certain amount of *α*-synuclein may be necessary to exert these pathological effects, as indicated by the genetic PD forms due to gene duplication and triplication.

## 5. LRRK2-Transgenic Models of PD

Mutations in leucine-rich repeat kinase 2 (LRRK2) are the most frequent cause of genetic PD [132, 133]. Clinical and neuropathological features of LRRK2-related PD are mostly indistinguishable from idiopathic PD [134, 135]. Transgenic overexpression of the entire human LRRK2 gene carrying the mutant Gly2019Ser in a mouse model results in abnormal dopamine signaling and increased levels of phosphorylated tau [136]. In this model, LRRK2 was expressed at high levels within the SVZ, OB, and the hippocampus which led to a significant reduction of proliferation and survival of newborn neurons within both neurogenic regions [137]. The morphology of newborn hippocampal neurons was markedly impaired with reduced dendrite length and spine density. It remains to be determined in the future whether common mechanisms are involved in the *α*-synuclein and the LRRK2 models. LRRK2, for example, has been shown to directly impair neurite outgrowth and dendritogenesis in* C. elegans* and in mouse neurons [138, 139].

Similar to *α*-synuclein, adult hippocampal neurogenesis was also studied in LRRK2-knockout mice [140]. Proliferation and survival of newborn neurons were not altered by deletion of endogenous LRRK2, but there was a significant increase of doublecortin-positive (DCX) neuroblasts with higher dendritic complexity in the knockouts. This pro-outgrowth effect may be either due to the direct effects of LRRK2 on neurite outgrowth or due to enhanced integration into the molecular layer of the dentate gyrus.

## 6. Clinical Implications of Compromised Neurogenesis

Adult newborn neurons have been proposed to exert different functions that partly overlap with premotor symptoms observed in PD.

Data from rodent studies indicate a function of newborn neurons in the adult hippocampus in depression. Depression is a frequent symptom in PD patients that often predates the onset of motor symptoms and was shown to have a high impact on quality of life in PD [141]. Serotonergic inputs to the hippocampus are decreased in PD-related depression (reviewed by [65]). There are indications that adult neurogenesis, on the other hand, is impaired in depression and that the effect of antidepressant therapy relies upon adult-generated neurons which led to the “neurogenic hypothesis of depression” [142–144]. A number of important studies have shown that alterations in adult neurogenesis are not the one single cause of depressive-like behavior; rather, the dentate gyrus, including the adult generation of newborn neurons, represents one part of a “mood-network” with other hippocampal subregions, amygdala, thalamus, the anterior pituitary, and other cortical and subcortical areas [145–148]. Data are mostly from preclinical models due to the methodological constraints of the investigation of adult neurogenesis in humans, but both MRI and post-mortem studies have shown reduced hippocampal volumes in major depressive disorder [149, 150]. Epidemiological studies and the presence of neurotransmitter imbalances in PD suggest depression as a specific nonmotor symptom in PD rather than a reactive pathogenesis due to impaired mobility [151]. Likewise, cognitive disturbances in PD (which are also related to pathology within the limbic system including the hippocampus) may be partly caused by alterations in adult neurogenesis. In fact, the most important function of adult hippocampal neurogenesis in rodents is the ability of memorizing two temporally related events (pattern separation [152, 153]). In light of this overlap with PD premotor symptoms and the known involvement of the hippocampus, changes in the plasticity of newborn neurons may contribute to the pathogenesis of depression and of cognitive decline but certainly need more investigation [154].

Regardless of the causal role of adult newborn neurons in PD pathology, strategies to reverse the observed neurogenesis defects might have therapeutic implications. In a recent paper, adult neurogenesis was rescued by chronic oral treatment with the selective serotonin reuptake inhibitor (SSRI) fluoxetine which is in routine use as antidepressant [99]. In fluoxetine-treated transgenic animals, proliferation, number of Sox2-positive progenitor cells, the number of DCX-positive neuroblasts, and the number of surviving newborn neurons were all restored to the level of fluoxetine-treated nontransgenic animals. As the levels of transgenic *α*-synuclein were unchanged upon treatment, the effects of fluoxetine rather made newborn neurons resistant to the deleterious effects of *α*-synuclein. This effect was paralleled by increased levels of the growth factors BDNF and GDNF which are both investigated in preclinical models of PD [155–157]. Fluoxetine treatment also showed marked benefits in a transgenic animal model of atypical PD expressing human *α*-synuclein under control of the myelin basic protein promoter [158]. SSRIs are often prescribed in depression including depression in PD; however, detailed studies about the clinical effect in PD patients are lacking. Dual modulation of the serotonergic pathway has been shown to accelerate the onset of antidepressant action on adult neurogenesis and may therefore also be tested in PD models [159].

Pharmacological screens have identified small molecules with a strong impact on adult neurogenesis [160], but the application in PD models has not been tested so far.

Physical activity, a known strong inducer of adult neurogenesis, was found to be another strategy to reverse PD-related alterations of adult neurogenesis as observed in the LRRK2-transgenic mouse model of PD [137]. Interestingly, deletion of the serotonin gene abolishes the proneurogenic effects of running indicating overlapping mechanisms and a causal role of serotonin in exercise-induced neurogenesis [161]. Different kinds of physical activity had positive effects on executive function and, to a limited degree, on cognition in PD patients and in lesion models of PD (reviewed by [162]). The activity-related rescue of adult neurogenesis may also be affected by disease pathology, as observed in a mouse model of Huntington's disease [163]. Therefore, investigation of the effects of physical activity on adult hippocampal neurogenesis in more PD models will be necessary.

In addition, there are speculations that counteracting inflammatory processes in PD may halt disease pathology. While epidemiological clinical studies may indicate a reduced PD risk after use of nonsteroidal anti-inflammatory drugs (NSAIDs) but have not been conclusive due to methodological difficulties [164, 165], it is widely accepted that neuroinflammation is involved in PD pathogenesis [166]. Neuroinflammatory activation is not confined to the substantia nigra but is found along with the progressing pathology of the disease [167]. This holds especially true for the olfactory bulb, where microgliosis is found in the olfactory bulb of PD patients [168] and mouse models [169]. The limbic system also shows an increased number of activated microglia in PD [170]. Notably, neuroinflammation induced by irradiation [171] or cortical injection of lipopolysaccharides [172] negatively regulates adult hippocampal neurogenesis. Moreover, the proinflammatory cytokine TNF-*α* impairs proliferation of neural progenitor cells in vitro [173]. Levels of TNF-*α* were found to be elevated in the serum of PD patients [174] and were associated with the presence of the nonmotor symptoms depression and anxiety in PD [175]. Thus, inflammatory processes in the neurogenic regions may contribute to the decline of neurogenesis in different transgenic animal models. A detailed analysis of inflammatory changes in the neurogenic regions of these models is still lacking but may represent one of the mechanisms contributing to the neurogenesis deficits. Adult neurogenesis itself is regulated by inflammatory activation and sophisticated studies showed both pro- and anti-inflammatory effects for different subtypes of microglia [176–178]. Interestingly, the modulation of adult neurogenesis by physical activity and enriched environment also seems to be dependent on microglial function [179, 180] which again underlines the need for a better understanding of microglial activation in the neurogenic niche in PD. Although the precise contribution of microglial activation to PD pathology is still elusive and may function as a multiplier of PD-associated neurodegeneration, an interaction between microglia and adult neurogenesis in the PD brain is likely.

## 7. Future Research

In summary, many studies in animal models have shown effects of PD pathology on the adult generation of newborn neurons, in part with conflicting results owing to different experimental conditions. Data on adult neurogenesis in human PD are still scarce but will be important to validate experimental findings. In the future, novel techniques will facilitate analysis of adult neurogenesis in animals and in patients [181, 182]. In addition, the discussed models of impaired neurogenesis in PD will serve as drug screening platform to validate drugs aimed at modifying the course of PD.
